# Exact Interior Reconstruction with Cone-Beam CT

**DOI:** 10.1155/2007/10693

**Published:** 2008-01-23

**Authors:** Yangbo Ye, Hengyong Yu, Ge Wang

**Affiliations:** ^1^Department of Mathematics, University of Iowa, Iowa City, IA 52242, USA; ^2^CT Laboratory, Biomedical Imaging Division, VT-WFU School of Biomedical Engineering, Virginia Tech, Blacksburg, VA 24061, USA

## Abstract

Using the backprojection filtration (BPF) and filtered backprojection (FBP) approaches, respectively, we prove that with cone-beam CT the interior problem can be exactly solved by analytic continuation. The prior knowledge we assume is that a volume of interest (VOI) in an object to be reconstructed is known in a subregion of the VOI. Our derivations are based on the so-called generalized PI-segment (chord). The available projection onto convex set (POCS) algorithm and singular value decomposition (SVD) method can be applied to perform the exact interior reconstruction. These results have many implications in the CT field and can be extended to other tomographic modalities, such as SPECT/PET, MRI.

## 1. INTRODUCTION

In 2002, an exact and efficient helical cone-beam reconstruction method was developed by Katsevich [1, 2], which is a significant breakthrough in the area of helical/spiral cone-beam CT. The Katsevich formula is in a filtered backprojection (FBP) format using data from a PI-arc corresponding to the so-called PI-segment. By interchanging the order of the Hilbert filtering and backprojection, Zou and Pan proposed a backprojection filtration (BPF) formula in the standard helical scanning case [3]. For important biomedical applications including bolus-chasing CT angiography [4] and electron-beam CT/micro-CT [5], our group obtained the first proofs of the general validities of both the BPF and FBP formulae in the case of cone-beam scanning along a general smooth scanning trajectory [6–9]. Other groups also made significant contributions along this direction [10–14].

The importance of performing exact image reconstruction from the minimum amount of data has been recognized for a long time. The first landmark achievement is the well-known fan-beam half-scan formula [15]. A recent milestone is the two-step Hilbert transform method developed by Noo et al. [16]. In their framework, an object image on a PI-line/chord can be exactly reconstructed if the intersection between the chord and the object is completely covered by a field of view (FOV). Very lately, Defrise et al. [17] proposed an enhanced data completeness condition that the image on a chord in the FOV can be exactly reconstructed if one end of the chord in the object is covered by the FOV. Inspired by the tremendous biomedical implications including local cardiac CT at minimum dose, local dental CT with high accuracy, CT guided procedures, nano-CT, and so on [18], we recently proved, using analytic continuation, that the interior problem can be exactly solved if a subregion in an region of interest (ROI) in the FOV is known [19, 20], while the conventional wisdom is that the interior problem does not have a unique solution [21].

A natural question is whether our exact interior reconstruction method [19, 20] can be extended to the interior reconstruction of a volume of interest (VOI)? Our positive answers will be provided here. The paper is organized as follows. In the next section, we summarize the relevant notations and key theorem. In the third section, we prove the feasibility of the exact 3D interior reconstruction using the BPF and FBP approaches, respectively. In the fourth section, we will present further ideas and conclude the paper.

## 2. NOTATIONS AND KEY THEOREM

The basic setting of our previous work is cone-beam scanning along a general smooth trajectory

(1) Γ={ρ(s)∣s∈ℝ}.

**Figure 1:**
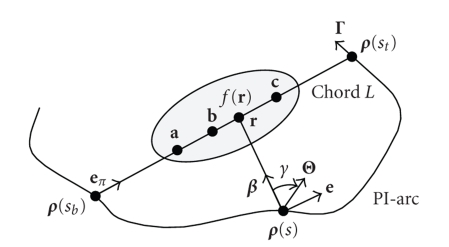
Basic setting for exact 3D interior reconstruction.

As shown in Figure 1, a generalized PI-line of $r\in {\mathbb{R}}^{3}$ is defined as the line through r and is intersecting the scanning trajectory at two points $\rho (s_{b})$ and $\rho (s_{t})$ on Γ with $s_{b}<s_{t}$, where $s_{b}=s_{b}(r)$ and $s_{t}=s_{t}(r)$ are the parameter values corresponding to these two points. At the same time, the PI-segment (also called a chord) L is defined as the segment of the PI-line between $\rho (s_{b})$ and $\rho (s_{t})$, the PI-arc is the part of the trajectory between $\rho (s_{b})$ and $\rho (s_{t})$, and the PI-interval is $\left[s_{b},s_{t}\right]$. Note that “PI” means “π.” Suppose that an object function f(r) is constrained in a compact support $\Omega \subset {\mathbb{R}}^{3}$. For any unit vector β, let us define a cone-beam projection of f(r) from a source point ρ(s) on the trajectory Γ by

(2) $$D_{f}(\rho (s),\beta ):=\int_{0}^{\infty }f(\rho (s)+t\beta )dt.$$

Then, we define a unit vector β(s,r) as the one pointing to r∈L from ρ(s) on the trajectory:

(3) $$\beta (r,s):=\frac{r-\rho (s)}{\left|r-\rho (s)\right|}.$$

We also need a unit vector along the chord:

(4) $$e_{\pi }:=\frac{\rho (s_{t})-\rho (s_{b})}{\left|\rho (s_{t})-\rho (s_{b})\right|}.$$

Note that the unit vector $e_{\pi }$ is the same for all r∈L. Our major finding can be summarized as the following theorem.

**Theorem 1**

*Assume that there are three points* a,b,c *on the chord* L *with* b *situating between* a *and* c. *Suppose that (i) the projection data* $D_{f}(\rho (s),\beta (r,s))$ *are known and* $D_{f}(\rho (s),-\beta (r,s))\equiv 0$, *both for any* $s\in [s_{b},s_{t}]$ *and for any* r *on the line-segment* $\bar{ac}$ (*and its small neighborhood*), *and* (ii) f(r) *is known on the line-segment* $\bar{ab}$. *Then, the function* f(r) *can be exactly and stably reconstructed on the line-segment* $\bar{bc}$.

We have several remarks on Theorem 1. Our condition (i) implies that the cone-beam projection data are both longitudinally and transversely truncated but the derivative ${\left(\partial /\partial q\right)D_{f}(\rho (q),\beta (r,s))|}_{q=s}$ is available for any $s\in [s_{b},s_{t}]$ and for any r on line-segment $\bar{ac}$ . This is the 3D interior reconstruction problem which does not have a unique solution according to the conventional wisdom [21]. Our condition (ii) demands prior information for the interior reconstruction which regularizes the ill-posedness of the interior reconstruction and make its solution accurate and robust. As discussed in our earlier paper [19], we may assume that the known data are on another subinterval of the line-segment $\bar{ac}$, or a union of such intervals. In practice, we may find that the function f(r) is known inside a subregion of the VOI, such as air around a tooth, water in a device, or metal in a semiconductor. Then, the exact interior reconstruction of the unknown parts of the VOI becomes feasible if their corresponding chords intersect with the known VOI.

## 3. PROOF OF THEOREM 1

### 3.1. Proof in the BPF framework

Our generalized BPF algorithm [6, 7] requires the backprojection of projection data derivative ${\left(\partial /\partial q\right)D_{f}(\rho (q),\beta )|}_{q=s}$ on a fixed chord and the inverse Hilbert transform along the 1D chord. Recall that the backprojection at r∈L can be expressed as [6, 7]

(5) $$\begin{array}{ll} g(r) & :=\int_{s_{b}}^{s_{t}}\frac{\partial }{\partial q}(D_{f}\left(\rho (q),\beta (r,s)\right)\\ & \;\;\;\;\;-D_{f}\left(\rho (q),-\beta (r,s))\right){|}_{q=s}\frac{ds}{\left|r-\rho (s)\right|}. \end{array}$$

Condition (i) implies that g(r) is available on the line-segment $\bar{ac}$. If we setup a local 1D coordinate system on the chord L, Theorem 1 can be reduced to the following 1D case.

**Theorem 2**

**Figure 2:**
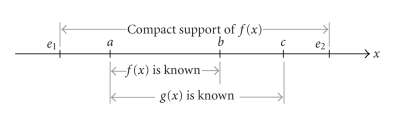
Setting for Theorem 2, where f(x) is supported on $\left[e_{1},e_{2}\right]$ and known on (a,b), while its Hilbert transform is known on (a,c).

*As shown in* Figure 2, *assume that* $e_{1}<a<b<c<e_{2}$ *and the 1D function* f(x) *is supported on the interval* $\left[e_{1},e_{2}\right]$. f(x) *can be exactly reconstructed on* (b,c) *if* (i) *the Hilbert transform* g(x) *of the function is known on* (a,c); (ii) f(x) *is known on* (a,b); *and* (iii) *the constant* $\int_{e_{1}}^{e_{2}}f(x)dx$ *is known*

Theorem 2 is exactly what we proved in our previous paper [19]. Hence, we complete the proof of Theorem 1 in the BPF framework. 

### 3.2. Proof in the FBP framework

For an arbitrary smooth scanning curve ρ(s) on the PI-interval $\left[s_{b},s_{t}\right]$ and any point r on the chord L from $\rho (s_{b})$ to $\rho (s_{t})$, the exact FBP reconstruction formula can be expressed as [8]

(6) $$\begin{array}{ll} f(r) & =-\frac{1}{2\pi^{2}}\int_{s_{b}}^{s_{t}}\frac{ds}{\left|r-\rho (s)\right|}\text{PV}\\ & \;\times \int_{0}^{2\pi }{\frac{\partial }{\partial q}D_{f}(\rho (q),\Theta (s,r,\gamma ))|}_{q=s}\frac{d\gamma }{\sin\gamma }, \end{array}$$

where “PV” represents a principal value integral, and Θ(s,r,γ) represents the filtering direction which is taken in the PI-segment direction and defined as cos⁡γβ+sin⁡γe with the unit directions β=β(r,s) and $e=\left(\left(e_{\pi }-\left(e_{\pi }\cdot \beta \right)\beta \right)/|e_{\pi }-\left(e_{\pi }\cdot \beta \right)\beta |\right)$, that is, Θ(s,r,γ) supposes a clockwise rotation in the plane determined by L and β(r,s), centered at ρ(s) with Θ(s,r,0)=β(r,s) (see Figure 1).

**Figure 3:**
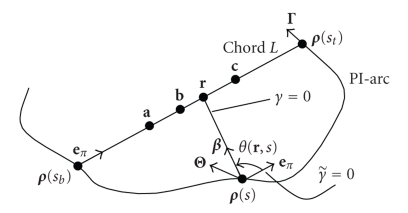
Variable change from γ to $\overset{˜}{\gamma }$.

For a fixed ρ(s), the filtering plane remains unchanged for all r∈L. As shown in Figure 3, we can change the variable γ to $\tilde{\gamma }$ so that the direction for $\tilde{\gamma }=0$ now points to the direction $e_{\pi }$, and the filtering direction is still specified clockwise. Let θ(r,s) denote the angle from $e_{\pi }$ ($\tilde{\gamma }=0$) to β(r,s). Then, (6) can be rewritten as

(7) $$\begin{array}{ll} f(r) & =-\frac{1}{2\pi^{2}}\int_{s_{b}}^{s_{t}}\frac{ds}{\left|r-\rho (s)\right|}\text{PV}\\ & \;\times \int_{-\pi }^{\pi }{\frac{\partial }{\partial q}D_{f}(\rho (q),\Theta (s,\overset{˜}{\gamma }))|}_{q=s}\frac{d\overset{˜}{\gamma }}{\sin(\overset{˜}{\gamma }-\theta (r,s))}. \end{array}$$

Note that Θ(s,r,γ) now is changed to $\Theta (s,\tilde{\gamma })$ which is independent of r∈L, and the value of θ(r,s) is negative. Our condition (i) implies that

(8) $$\text{PV}\int_{\theta (a,s)}^{\theta (c,s)}{\frac{\partial }{\partial q}D_{f}(\rho (q),\Theta (s,\overset{˜}{\gamma }))|}_{q=s}\frac{d\overset{˜}{\gamma }}{\sin(\overset{˜}{\gamma }-\theta (r,s))}$$

is known for any $s\in [s_{b},s_{t}]$ and for any r on the line-segment $\bar{ac}$.

**Figure 4:**
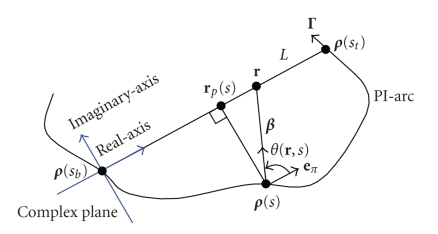
Complex coordinate system for the analytic continuity.

To reconstruct f(r) on the line-segment $\bar{bc}$, we need to know that

(9) $$\begin{array}{ll} h(r) & =-\frac{1}{2\pi^{2}}\int_{s_{b}}^{s_{t}}\frac{ds}{\left|r-\rho (s)\right|}\text{PV}\\ & \;\times \int_{\begin{array}{l} \overset{˜}{\gamma }\in [-\pi ,\pi ],\overset{˜}{\gamma }\notin [\theta (a,s),\theta (c,s)] \end{array}}\\ & {\;\;\times \frac{\partial }{\partial q}D_{f}(\rho (q),\Theta (s,\overset{˜}{\gamma }))|}_{q=s}\frac{d\overset{˜}{\gamma }}{\sin(\overset{˜}{\gamma }-\theta (r,s))} \end{array}$$

for r on the line-segment $\bar{bc}$. In fact, the inner integral of (9) is an ordinary integral for r on the line-segment $\bar{ac}$. Let $r_{p}(s)$ denote the point on L such that $\left(r_{p}(s)-\rho (s)\right)$ is perpendicular to L, as shown in Figure 4. Then, $\text{sin}\;(\overset{˜}{\gamma }-\theta (r,s))=\text{sin}\;\overset{˜}{\gamma \;}\text{cos}\;(\theta (r,s))-\text{cos}\;\overset{˜}{\gamma }\;\text{sin}\;\left(\theta (r,s)\right)$ with

(10) $$\begin{array}{ll} \text{sin}\left(\theta (r,s)\right) & =\frac{-\left|r_{p}(s)-\rho (s)\right|}{\left|r-\rho (s)\right|},\\ \text{cos}\left(\theta (r,s)\right) & =\frac{\varepsilon \left|r-r_{p}(s)\right|}{\left|r-\rho (s)\right|}, \end{array}$$

where ε=1 if r is on the $\rho (s_{t})$ side of $r_{p}(s)$ and ε=−1 if r is on the $\rho (s_{b})$ side of $r_{p}(s)$. If we use complex plane coordinates (see Figure 4) with the origin $\rho (s_{b})$ and the positive direction from $\rho (s_{b})$ to $\rho (s_{t})$, then we have

(11) $$\cos\left(\theta (r,s)\right)=\frac{r-r_{p}(s)}{\left|r-\rho (s)\right|}.$$

Then, h(r) in (9) becomes

(12) $$\begin{array}{ll} h(r) & =-\frac{1}{2\pi^{2}}\int_{s_{b}}^{s_{t}}ds\int_{\begin{array}{l} \overset{˜}{\gamma }\in [-\pi ,\pi ],\overset{˜}{\gamma }\notin [\theta (a,s),\theta (c,s)] \end{array}}\\ & {\;\;\;\;\;\;\times \frac{\partial }{\partial q}D_{f}(\rho (q),\Theta (s,\overset{˜}{\gamma }))|}_{q=s}\\ & \;\;\;\;\;\;\times \frac{d\overset{˜}{\gamma }}{\text{sin}\;\overset{˜}{\gamma }\left(r-r_{p}(s)\right)+\text{cos}\;\overset{˜}{\gamma }\left|r_{p}(s)-\rho (s)\right|}. \end{array}$$

Note that the denominator under $d\tilde{\gamma }$ is nonzero for r on $\bar{ac}$ in the real axis. Therefore, if we replace r by z in (12), h(z) is an analytic function on the complex plane with cuts along the real axis from −∞ to a and from c to +∞. As a result, we can always take derivatives with respect to z under integration on the right side of (12), and the proof follows the same arguments as in the proof of Cauchy’s integral theorem.

By condition (ii), f(r) is known on the line-segment $\bar{ab}$. Hence,

(13) $$\begin{array}{ll} f(r) & =-\frac{1}{2\pi^{2}}\int_{s_{b}}^{s_{t}}\frac{ds}{\left|r-\rho (s)\right|}\text{PV}\\ & \;\times \int_{\theta (a,s)}^{\theta (c,s)}{\frac{\partial }{\partial q}D_{f}(\rho (q),\Theta (s,\overset{˜}{\gamma }))|}_{q=s}\\ & \;\;\;\;\times \frac{d\overset{˜}{\gamma }}{\text{sin}(\overset{˜}{\gamma }-\theta (r,s))}+h(r) \end{array}$$

is known for r on the line-segment $\bar{ab}$. Assumption (i) and (8) imply that the first term on the right side of (13) is also known. Therefore, h(z) is known for any z on the line-segment $\bar{ab}$. Then, by analytic continuation, h(z) on the line-segment $\bar{bc}$ can be uniquely determined by its value on the line-segment $\bar{ab}$. That is, (9) is known for r on the line-segment $\bar{bc}$. By assumption (i), (8) is known for r on the line-segment $\bar{bc}$. This gives us the exact and stable reconstruction of f(r) on the line-segment $\bar{bc}$ by (7). That is, Theorem 1 is proved in the FBP framework.

We have two remarks for the above proof. First, these arguments work for the generalized PI-line filtering direction only. If the filtering direction is not in the PI-line direction, neighboring points on the same PI-line will require different filtering integrals. In this case, currently we do not know how to link a filtering integral to another filtering integral for the interior reconstruction. Second, the translation from θ(r,s) to $r-r_{p}(s)$ is a crucial step. Without such a step, one cannot deal with the effect of the outer integral in (9). With that change, (12) becomes manageable because r only appears in the inner integral.

## 4. DISCUSSIONS AND CONCLUSION

While we have proved that the exact and stable 3D interior reconstruction is feasible from data focusing on a VOI and collected along a general smooth scanning trajectory, we believe that our results can be also extended to the case of discontinuous scanning trajectories. The general exact cone-beam reconstruction results were reported for both continuous and discontinuous trajectories [6–8, 12, 13]. Similarly, we can use the same tricks such as in [12, 13] to formulate more general results. We will elaborate this type of ideas further in the future, such as for triple-source cone-beam CT [22, 23].

Because the closed-form method for analytic continuity is unavailable, we adapted a projection onto convex set (POCS) method [19] and a singular value decomposition (SVD) [20] method for exact 2D interior reconstruction, and these methods can be further adapted for exact 3D interior reconstruction and should have the same noise characteristics. Moreover, the BPF and FBP formulations will lead to different numerical implementations for exact 3D interior reconstruction when an analytic continuity method is given. According to our theorem, the function value of f(r) must be known in some subregions of a VOI to be reconstructed. For practical applications and further research, we may use and add other constraints or prior information into the interior reconstruction process such as an iterative reconstruction procedure. These additional constraints may be included but not be limited to mean and other moment values, histogram features, maximum/minimum values of subregions or involved components, and low-resolution images related to the VOI (subregions or neighbors). Even if we do not know the exact function value of f(r) in a subregion or we do not necessarily need exact reconstruction, we can still utilize our analytically obtained guidelines to construct approximate reconstruction algorithms.

In addition to CT-specific interior reconstruction techniques, we recognize that our approach for interior reconstruction can be readily applied for MRI, SPECT, PET, and other geometric optic-based imaging modes. Furthermore, we feel that our exact interior reconstruction results can be extended into the case of the exponential attenuated radon transform [24, 25]. Specifically, we can use iterative algorithms to produce superior images in the same spirit of the exact interior CT reconstruction. Our general hypothesis is that we can start with a generalized Hilbert transform of attenuated radon data and reach similar conclusions by analytic continuation. While analytic algorithms may be developed for the uniformly attenuation SPECT/PET, iterative algorithms (e.g., POCS) should be feasible for exact 3D interior SPECT/PET reconstruction in the case of non-uniformly attenuation background.

In the CT field, the most popular imaging model assumes a monochromatic source and a motionless subject. Theorem 1 in this paper is also based on the same assumption. However, our results are also relevant to polychromatic and/or dynamic imaging. By utilizing truly local data instead of global data, we may achieve better temporal resolution, higher image contrast, less image artifacts, and so on. This aspect seems deserving more research efforts as well.

In conclusion, using the BPF and FBP approaches, respectively, we have proved that the 3D exact interior reconstruction is feasible from both longitudinally and transversely truncated data collected along any general scanning trajectory only through an internal VOI. The major mathematical tool which we have used is the analytic continuation theory. Our previous reconstruction algorithms for exact 2D interior reconstruction can be directly applied in the 3D case. Our results can take other mathematical forms, can be extended to other imaging fields, and have tremendous application potentials. We are actively working to realize selected possibilities.
